# Thymus and autoimmunity

**DOI:** 10.1007/s00281-021-00842-3

**Published:** 2021-02-03

**Authors:** Alexander Marx, Yosuke Yamada, Katja Simon-Keller, Berthold Schalke, Nick Willcox, Philipp Ströbel, Cleo-Aron Weis

**Affiliations:** 1 Institute of Pathology, University Medical Centre Mannheim, University of Heidelberg, Theodor-Kutzer-Ufer 1-3, 68167 Mannheim, Germany; 2 Department of Diagnostic Pathology, Kyoto University Hospital, Kyoto, 606-8507 Japan; 3 Department of Neurology, Bezirkskrankenhaus, University of Regensburg, 93042 Regensburg, Germany; 4 Neurosciences Group, Nuffield Department of Clinical Neurology, Weatherall Institute of Molecular Medicine, John Radcliffe Hospital, University of Oxford, Oxford, UK; 5 Institute of Pathology, University Medical Center Göttingen, University of Göttigen, 37075 Göttingen, Germany

**Keywords:** Thymus, Myasthenia gravis, Tuft cells, Myoid cells, AIRE, FEZF2

## Abstract

The thymus prevents autoimmune diseases through mechanisms that operate in the cortex and medulla, comprising positive and negative selection and the generation of regulatory T-cells (Tregs). Egress from the thymus through the perivascular space (PVS) to the blood is another possible checkpoint, as shown by some autoimmune/immunodeficiency syndromes. In polygenic autoimmune diseases, subtle thymic dysfunctions may compound genetic, hormonal and environmental cues. Here, we cover (a) tolerance-inducing cell types, whether thymic epithelial or tuft cells, or dendritic, B- or thymic myoid cells; (b) tolerance-inducing mechanisms and their failure in relation to thymic anatomic compartments, and with special emphasis on human monogenic and polygenic autoimmune diseases and the related thymic pathologies, if known; (c) polymorphisms and mutations of tolerance-related genes with an impact on positive selection (e.g. the gene encoding the thymoproteasome-specific subunit, *PSMB11*), promiscuous gene expression (e.g. *AIRE*, *PRKDC*, *FEZF2*, *CHD4*), Treg development (e.g. *SATB1*, *FOXP3*), T-cell migration (e.g. *TAGAP*) and egress from the thymus (e.g. *MTS1*, *CORO1A*); (d) myasthenia gravis as the prototypic outcome of an inflamed or disordered neoplastic ‘sick thymus’.

## Introduction

**Fig. 1:**
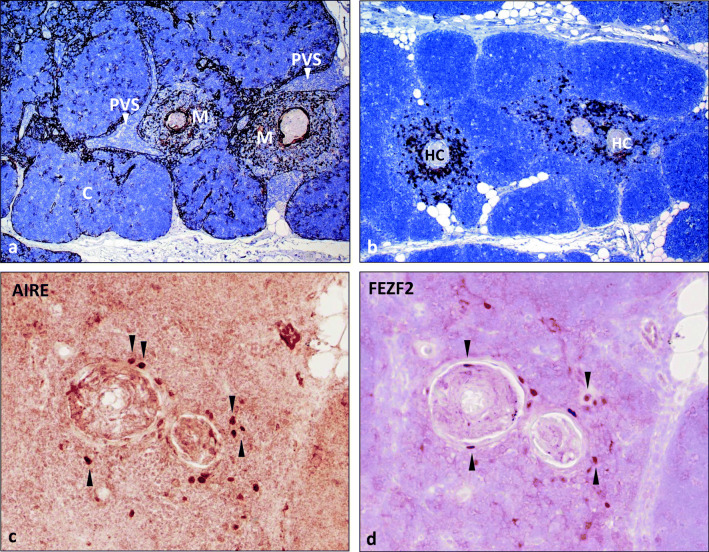
Normal thymus. **a** Labelling of thymic epithelial cells with an anti-keratin 19 antibody reveals three thymic compartments: cortex (C), medulla (M) and perivascular space (PVS, arrowheads). **b** Moderate content of CD20^+^ B-cells around Hassall corpuscles (HC) in the medulla (M) of an adult thymus (40 years of age). **c**, **d** Nuclear AIRE and FEZF2 expression in medullary thymic epithelial cells around two HCs (sequential staining of the same section). Many cells with AIRE/FEZF2 co-expression. Arrowheads highlight cells that stain exclusively for either AIRE or FEZF2. Immunoperoxidase

The thymus generates responsive T-cells from immature precursors (together called ‘thymocytes’) as key players in a functional adaptive immune system. It also prevents human autoimmune diseases (HAIDs) through both negative selection (by which most autoreactive α/β-T-cells are deleted [1, 2]) and generation of FOXP3^+^ regulatory T-cells (Tregs) [3–6] that restrain those autoreactive T-cells that inevitably escape negative selection and seed the periphery [7, 8]. Thymic tolerogenic mechanisms require thymic epithelial cells (TECs), dendritic cells (DCs) and B-cells and involve the cortex, medulla and perivascular spaces (PVS) (Fig. 1).

Monogenic HAIDs have been crucial to identifying key contributors to tolerance, e.g. the autoimmune polyendocrinopathy-candidiasis-ectodermal dystrophy syndrome (APECED, alias autoimmune polyglandular syndrome type 1, APS1) due to autoimmune regulator (*AIRE*) mutations [9, 10], the immunodysregulation polyendocrinopathy and enteropathy X-linked (IPEX) syndrome) resulting from *FOXP3* mutations [11] and ‘leaky’ (subtotal) immunodeficiency syndromes due to primary T-cell or stromal cell developmental defects [12, 13]. Parallel studies in mouse models helped to elucidate underlying mechanisms and their non-redundancy [6, 14–16]. In many sporadic polygenic HAIDs, the role of the thymus is difficult to prove, since disorders in tolerogenesis are often subtle and need complementary genetic, epigenetic, hormonal or environmental cues [17]. ‘Primary’ inflammatory and neoplastic disorders of the thymus in myasthenia gravis (MG) have highlighted thymic abnormalities that correlate with autoimmunity in humans [18].

## The thymic medulla and autoimmunity

### AIRE expression in the thymic medulla

Identifying *AIRE* mutations as the cause of the APECED syndrome [9, 10] has deepened the understanding of negative selection. AIRE also supports the generation of antigen-specific natural Tregs in mice [15] and humans [19]. In humans, AIRE is expressed in the nuclei of rare mTECs mainly in the vicinity of Hassall corpuscles (Fig. 1). Since APECED thymic tissue is not available, AIRE’s function has been elucidated through mouse models, although they do not fully mirror the clinical features of human APECED [20–22].

#### AIRE and negative selection

AIRE is a transcriptional modulator that is mainly expressed in MHCII^high^ mTECs. Their development from AIRE^--^, MHCII^low^ precursors requires RANK/RANKL and CD40/CD40L signalling that, in turn, depends on thymopoiesis [23–25]; when it is deficient, thymic AIRE expression is therefore also missing, as in some primary T-cell immunodeficiencies. In mTECs, AIRE is part of a multimeric complex including transcription factors, enhancers and DNA repair molecules [26] and drives the low-level ‘promiscuous gene expression’ (pGE) of more than 3000 tissue-restricted self-antigens (TRAs) [16], comprising about 40% of all TRAs expressed in the thymus [27]. Presentation of MHC-bound, thymoproteasome-processed TRA-peptides [28] by mTECs deletes any maturing thymocytes with high affinities for these complexes [14, 16] or diverts them into the Treg lineage [29]. By contrast, cleavage in autophagic vacuoles is needed for peptide loading onto MHCII molecules [30], and blocking autophagy in mice elicits autoimmune colitis and multi-organ inflammation [31].

To achieve pGE, AIRE is preferentially recruited to promoters with low levels of H3K4 methylation [32]. It also induces topoisomerase II-dependent double-strand breaks, the editing and splicing of RNA [26, 33, 34]; promotes the release of stalled RNA polymerase-II [35] and enhances the binding of super-enhancers to ‘orderly relaxed’ chromatin [36, 37]. Furthermore, AIRE-dependent RNAs are preferentially stabilised through the 3′ end processing complex that generates short 3′ UTRs and protects against miRNA-mediated degradation [38]. In the mouse, the spectrum of AIRE-driven TRAs is further broadened by cooperation of AIRE with the transcription factor, Fezf2, and the chromatin modulator, Chd4 [39]. On the other hand, the expression of AIRE and its target genes is suppressed by oestrogens, which may explain the gender bias of several autoimmune diseases [40].

Since any single AIRE-dependent TRA is stochastically expressed in only 1–3% of mTECs at a given time point [41, 42], thymocytes must be highly mobile to ensure sufficiently systematic screening throughout the thymic medulla for full tolerance induction [43]. Indeed, thymocyte motility defects can associate with autoimmunity [44].

Thymic DCs are also motile and can cross-present TRA-derived MHC/peptide complexes, enhancing tolerance induction to TEC-derived TRAs [8, 45–47], especially any derived from AIRE-independent TRAs [48].

AIRE expression in cells other than mTECs: AIRE is also expressed at lower levels in minority subsets of (i) murine and human thymic B-cells [49–52], (ii) CCR7^+^ PDL1^--^CD127^+^ medullary thymic DCs [53, 54] and (iii) rare extrathymic DCs [55]. While B-cells and DCs are important for tolerance induction, the relevance of AIRE expression in a few of each is less clear: it might even be linked to the maturation of thymic and peripheral DCs [53].

#### AIRE and Treg development

AIRE is involved in the positive selection of a subset of self-antigen-specific Tregs, whether directly through AIRE^+^ mTECs [15] or indirectly by their transfer of MHCII/TRA peptide complexes to AIRE^--^ DCs [56], using different molecular transfer mechanisms for cell surface and cytosolic proteins [47]. It is unknown why some AIRE-dependent TRAs preferentially induce Treg development [29, 57], whereas others induce deletion [58].

#### Human AIRE-related autoimmune features

Patients with inactivating *AIRE* mutations develop APECED due to autoimmune T- and B-cell responses that damage many organs, preferentially the adrenal cortex and parathyroid glands [20]. In contrast to AIRE-deficient mice [21], nearly all APECED patients show neutralizing autoantibodies to type I interferons and TH17 interleukins [59–61], and loss of Th17 and Th22 cells [62], which correlate with the characteristic mucocutaneous candidiasis, which is often the first sign of APECED [62, 63]. Their autoantibodies to Th17-directed cytokines interfere with macrophage activation, whereas others (e.g. against IL-22) reportedly protect against type 1 diabetes (T1D) [64]. APECED patients also variably share autoantibodies with other HAIDs, including cancer tissue antigens [65], suggesting a role of the thymus in anti-tumour immunity [66, 67].

*AIRE* polymorphisms have been associated with sporadic vitiligo [68] and rheumatoid arthritis (RA) [69] but not with other common autoimmune diseases such as T1D [70]. Mutations of genes that encode ‘*AIRE partners*’ (e.g. mutations of *PRKDC* [71]) can cause APECED-like syndromes.

#### Pathology

The features of thymi in APECED patients are unknown. AIRE expression deficits in humans occur in thymomas (see below) and primary immunodeficiencies.

### FEZF2 and CHD4 expression in mTECs

The second gene identified to drive TRA expression in the thymus is the transcription factor ‘forebrain expressed zinc finger 2’ (*Fezf2*) [72]. It is mainly expressed in the brain [73, 74], also close to Hassall corpuscles in the human thymus, and in a subset of mTECs, some being AIRE^+^ too [39] (Fig. 1). Fezf2 regulates the expression of about 400 TRAs that are distinct from those regulated by AIRE [34]. About 1000 TRAs are co-regulated by either Fezf2 and AIRE [34] or Fezf2 and Chd4 [39]. Transplantation of Fezf2^−/−^ thymi into nude mice elicits organ infiltrates and autoantibodies that are different from those in AIRE^−/−^ mice [14, 72].

Recently, chromodomain helicase DNA-binding protein 4 (Chd4) was identified as the first Fezf2-interacting protein [39]. It is a broadly expressed chromatin modulator that elicits histone modifications of its target genes distinct from those in AIRE-induced genes [39]. Chd4 and Fezf2 cooperatively regulate the expression of more than 25% of Fezf2-dependent genes by modifying the chromatin state around them, while Chd4’s co-regulation of 30% of AIRE-dependent genes [39] involves super-enhancers [34]. In mice, Chd4 induces the expression of a small set of unique genes [39].

#### Human *FEZF2* and *CHD4*-related autoimmune features

As yet, there is no genetic or pathologic evidence directly to incriminate FEZF2 or CHD4 in either sporadic HAIDs or syndromes analogous to APECED. However, some Fezf2-dependent TRAs identified in mice are autoantigens in humans, including aquaporin 8 (AQP8) in Sjögren syndrome [75] and transthyretin (TTR) in juvenile idiopathic arthritis (JIA) [76]. Some of the Chd4-(co-)regulated genes in mice encode human autoantigens, including TSHR, the key autoantigen in Graves’ disease [77, 78].

### Other autoimmune risk polymorphisms involving mTECs

Some risk polymorphisms for sporadic HAIDs operate in the thymus, e.g. those in the AIRE-driven *INS/insulin* promoter that associate with insulin expression levels in the thymus rather than the pancreatic islets and, inversely, with the risk of developing T1D [79, 80] even among APECED patients [81]. Similarly, a polymorphism in the IRF8-binding site in the promoter of the AIRE-driven gene encoding the acetylcholine receptor (AChR) α-subunit has been linked to reduced AChR expression in the thymus and the risk of very early–onset myasthenia gravis [82]. Analogous scenarios apply to Graves’ disease [77], autoimmune myocarditis [83] and central nervous system autoimmunity (see A. Handel, this fascicle).

### Hassall corpuscles, thymic tuft cells, thymic myoid cells and autoimmunity

Like thymic tuft cells, the squamoid cells that constitute Hassall corpuscles (HCs) in the human medulla (Fig. 1) and inconspicuous aggregates in the murine thymus [84] are terminally differentiated mTECs [85–87]. Thymic myoid cells are rare skeletal muscle-like cells occurring close to HCs [88].

Hassall corpuscles arise from AIRE^+^ MHCII^high^ mTECs under the influence of thymocyte-dependent lymphotoxin signals [87]. Terminal mTEC differentiation is accompanied by downregulation of AIRE, MHCII and CD80/CD86 and upregulation, e.g. of KRT10, involucrin, desmogleins and serine protease inhibitor (SPINK5), characteristic of terminally mature cutaneous keratinocytes [34, 86]. Despite the downregulation of AIRE, squamoid mTECs still express many AIRE-dependent and independent TRAs before dying inside HCs [48]. HCs supposedly promote tolerance in two ways: through transfer of TRAs to nearby DCs for cross-presentation [34] and through secretion of thymic stromal lymphopoietin (TSLP) that induces CD80/CD86 on MHCII^+^ DCs that, in turn, promotes Treg development in the presence of IL-2 [89, 90]. The TSLP pathway may not operate in mice, whose squamoid mTECs lack TSLP expression [85].

Thymic tuft cells (TTCs) in mice likely represent the rare microvillous mTECs [91]. They arise from AIRE^+^ and AIRE^--^ mTECs [85, 86] and partially resemble the chemosensory tuft (‘brush’) cells that were first identified in mucosal tissues [92, 93] and meanwhile in many other organs [94]. Like other tuft cells but unlike other TECs, TTCs develop under the control of the transcription factor, POU2F3, and express many tuft cell markers, including IL-25, the protein kinase DCLK1, pro-inflammatory cyclooxygenases [95], proteins involved in acetylcholine metabolism (e.g. ChAT) and taste transduction (e.g. Trpm5); yet, they do not show pGE [85, 86]. On the other hand, TTCs specifically express MHCII and CD74 that are involved in antigen presentation; they also induce tolerance against IL-25 that is lost in TTC-deficient mice [86], though its breadth and mechanisms are currently unknown.

Thymic myoid cells (TMCs) are evolutionarily conserved, non-innervated mesenchymal cells that resemble myoblasts or myotubes [88] and occur in the normal medulla (i.e. near HCs, AIRE^+^ and FEZF2^+^ mTECs, POU2F3^+^ thymic tuft cells and thymic B, T and DCs). Their origins [96, 97] and kinship to mTECs with a ‘myoid phenotype’ [54] are unclear. In contrast to mTECs, TMCs express AChR in its *native* confirmation [98] that is exclusively recognised by MG patients’ autoantibodies: indeed, they are the only cells outside the muscle to express this key target autoantigen, as well as titin [99] and ryanodine receptors (RyRs) [100]. Since TMCs are MHCII^--^ [98], they may contribute to tolerance through transfer of muscle self-antigens to DCs for cross-presentation to T-cells [18]. So may isolated AChR subunits or other muscle proteins that are also expressed by mTECs [54, 98, 101], suggesting that immune tolerance to skeletal muscle has been a high priority during evolution.

#### Human autoimmune features related to HCs, TTCs and TMCs

Impaired TEC/thymocyte crosstalk leads to lack of AIRE^+^ mTECs and of the HCs that they generate, possibly increasing risks of HAIDs: many primary T-cell immunodeficiency syndromes, e.g. hypomorphic defects of RAG-1, and some inborn errors of thymic stroma development result in lymphocyte-poor, AIRE^--^ and HC-deficient thymic rudiments (‘thymic dysplasia’) and may associate with HAIDs (see R. Bachetta and F. Dhalla in this fascicle). So does trisomy 21, with three *AIRE* alleles, enlarged HCs and increased risks of T1D, but the mechanisms involved are controversial [102–104]. The decline of HC numbers during aging [105] results from a decline of haematopoietic and epithelial cell functions [106] and may contribute to the increased prevalence of some HAIDs in the elderly [107]. Thymic tuft cell deficiency has not been reported in humans. Combined deficiencies of TMCs, HCs and AIRE expression in thymomas are detailed below.

### Hematopoietic cells in the thymic medulla and autoimmunity

Thymic dendritic cells are classified as CD8α^+^Sirpα^--^ conventional DCs (cDC1s in mice and CD141^+^ cDCs in humans), CD8α^--^Sirpα^+^ DCs (cDC2s, including a monocyte-derived CD14^+^ DC subset [108]) and plasmacytoid DCs (pDCs) [108–110]. cDC1s are generated intrathymically from immature precursors recruited to the thymus by mTEC-derived CCL21s [111], while other DCs are attracted from the periphery as mature cells [112, 113] by mTEC-derived chemokines, some of which require toll-like receptor 9 (TLR9)/MYD-88 signalling for production [108]. AIRE-dependent mTECs secrete XCL1 [114] that attracts cDCs and facilitates their acquisition of promiscuously expressed antigens from mTECs [45–47, 115], while pDCs essentially present peripheral antigens [113]. Antigen transfer from mTECs to DCs is key for the cross-presentation of promiscuously expressed antigens for negative selection [8, 45, 115] and the generation of Tregs [47, 114, 116]. Compared with mTECs and medullary B-cells, DCs show the highest expression levels of *HLA* genes but low levels of *TRA* genes [50, 53].

Thymic B-cells occur in the medulla from foetal life onwards [117]. Their abundance increases with age. Thymic AIRE^+^ and AIRE^--^ B-cells play a role in deletional tolerance: following activation through autoreactive T-cells and CD40 signalling, B-cells express AIRE together with a set of TRAs and present MHCII/TRA-peptide complexes, and so specifically delete the autoreactive T-cells that activated them [52, 118]. In humans, 5% of thymic B-cells express AIRE [49, 50, 119]. Since their set of AIRE-dependent TRAs is different from that in mTECs, thymic B-cells may delete T-cells with distinct self-reactive specificities [49, 50, 119] or help to divert them into the Treg lineage [120–122].

#### Regulatory T-cells

A minority of developing CD4^+^ T cells with α/β-T-cell receptors (TCR) develops towards the Treg lineage if recognizing self-peptide/MHCII complexes with intermediate affinities [123]. Thus, the TCR repertoire of thymus-derived Tregs (tTregs) is skewed towards recognizing self-antigens compared with conventional CD4^+^ helper T-cells (TH-cells) [124]. Tregs constitute about 10% of all CD4^+^ T-cells, of which 80–90% are tTregs; peripheral Tregs (pTregs) arise from mature, conventional CD4^+^ T-cells [125]. The development of tTregs starts in the cortex: in the presence of TCR and IL-2/STAT3 signalling, the transcription factor, SATB1, binds to closed DNA regions in cortical CD4^+^CD8^+^ thymocytes to initiate chromatin opening. A defect at this level elicits Treg deficiency and autoimmunity [126]. Subsequently, CD4^+^ CD8^--^ single positive thymocytes develop in the medulla through further epigenetic modifications, establishment of the ‘Treg-specific demethylated region, TSDR’ [127] and binding of transcription factors (e.g. RUNX1, CBFFB, ETS1, FOXO1 and 3) that drive expression of *CD25*, *FOXP3*, *CTLA4* and other ‘Treg signature genes’ [128]. Cues from Hassall corpuscle-instructed medullary DCs [89] and AIRE^+^ mTECs [15, 19] in the presence of MHCII, CD80/86 and IL-2 [90] control the abundance, antigen-specificity and suppressive competence of these tTregs. Once expressed, FOXP3 maintains survival and function of Tregs through driving target gene expression (e.g. of *CD25/IL2RA* and *CTLA4*) or suppression of pro-inflammatory *IL2* and *IFNG*) [123]. Recently, two developmental pathways leading to tTregs with distinct target specificities were described [129] and may be present in the human thymus [54].

#### Human autoimmune features related to hematopoietic cells

Thymic DC numbers do not change much during ageing [130], but proinflammatory genes (e.g. *LIF*, *IL6*) are increasingly expressed and may contribute to involution [131, 132], though with no proven link to HAIDs.

In B-cells, the declining transcription with age of *AIRE* and rare *TRA* genes, including *TTN* (encoding the muscle protein, titin), has been linked to the commoner occurrence of some HAIDs including MG in the elderly [49, 107].

Severe defects of Tregs occur in monogenic autoimmune diseases. For example, IPEX syndrome (analogous to murine scurfy syndrome [3]) results from different mutations across the *FOXP3* gene [133], showing that Tregs are indispensable to prevent T1D (even perinatally), inflammatory bowel disease and allergies, although the clinical variability of IPEX correlates poorly with the type of *FOXP3* mutation [134]. Other mutations in *CD25*, *CTLA4*, *LRBA*, *BACH2* and *STAT3* cause ‘IPEX-like syndromes’ due to Treg dysfunction. Differences in their expression, e.g. in follicular TH- and B-cells, may contribute to clinical differences between these ‘Tregopathies’ [135] (see R. Bacchetta in this fascicle). In addition, genetic variants in *Treg-related loci* associate with some common sporadic autoimmune diseases [128, 136].

#### Pathology

The thymus in an IPEX patient showed dysplasia, i.e. lack of lymphoid cells and Hassall corpuscles [137]. These changes are likely secondary, resembling those in scurfy mice, where severe thymic atrophy likely results from the cytokine storm and lymphoproliferation that develop in the absence of Tregs [138].

## The thymic cortex and autoimmunity

The thymic cortex provides the microenvironment for positive selection of conventional T-cells and early Tregs. Thus, its cTECs generate distinctive self-peptides via a unique set of proteases: (a) to select CD8^+^ thymocytes, cytosolic peptides are generated for presentation on MHCI molecules by the cortex-restricted ‘thymoproteasome’, with its unique Beta5t subunit (encoded by *PSMB11*) [28]; (b) to select CD4^+^ thymocytes, MHCII molecules in cTECs are loaded inside LAMP2^+^ endosomes with various endogenous self-peptides generated using cathepsin L and the thymus-specific serine protease, TSSP (encoded by *CTSL* and *PRSS16*, respectively) [139]. Autophagy in cTECs is one source of such MHCII:peptide complexes [30]; they also owe their persistence on the cTEC surface to CD83-dependent blockade of MACH-8-mediated trafficking there [140, 141]. This positive selection clearly depends on some crucial survival signals for nascent T-cells delivered via their TCRs, available co-receptors and downstream molecules (such as the tyrosine kinase, ZAP70) that transmit TCR signals [142–144]. Once positively selected, thymocytes upregulate chemokine receptors (e.g. CCR7) and must migrate to the medulla along chemotactic gradients [145, 146] for proper establishment of central tolerance [147].

### Human autoimmune features related to thymic cortical dysfunction

Homozygosity for the rs54457782 SNP of *PSMB11* has been associated with altered B5t function of the protein in cTECs and an elevated risk of Sjögren syndrome in one study [148].

Deletion of *Prss16* in cTECs clearly protects NOD mice against T1D, presumably by affecting processing of pancreatic islet cell TRAs [149]. A role of *PRSS16* in HAIDs has not been proven.

Polymorphisms of the C-type lectin *CLEC16A* gene show associations with T1D, multiple sclerosis (MS), systemic lupus (SLE), celiac disease, RA and JIA. Mouse studies implicate CLECA16’s impact on autophagy in cTECs or mTECs and thus on the repertoire of MHCII/self-peptide complexes for CD4^+^ T-cell selection [150].

In a similar scenario, ‘autoimmunizing positive selection’ (complemented by defective negative selection) might be operative in thymomas (see below), and in patients with *ZAP70* mutations: while inactivating mutations of *ZAP70* cause severe immunodeficiency, hypomorphic mutations lead to positive selection of autoreactive thymocytes [142]. Since attenuated ZAP70 signalling also attenuates negative selection and selection of Tregs, autoimmunity arises [151], leading to bullous pemphigoid, colitis and proteinuria in patients [152].

Nucleotide variants of *TAGAP* that encode a thymocyte GAP protein are associated with various HAIDs, likely reflecting attenuated thymocyte migration from the cortex to the medulla [44].

Finally, associations of SNPs of *SATB1* with colitis, psoriasis and MS have been linked to SATB1’s role in Treg development in the thymic cortex [126].

### Pathology

Expansion of the thymic cortex at the near-total expense of medullary regions is typical of thymomas (see below). Conversely, secondary cortical atrophy can result from the chronic re-entry of activated peripheral T-cells into the thymus in HAIDs [153]. Cortical atrophy as a facet of thymic involution during aging is thought to increase the risk of HAIDs [154].

## PVS and autoimmunity

The third thymic compartment with relevance for autoimmunity is the epithelial-free perivascular space (PVS) (Fig. 1). It surrounds vessels that enter the thymus through the septa between cortical lobules up to the corticomedullary junction (CMJ) [155]. The PVS extends between the basal membranes of the outermost epithelial cells of thymic lobes to those of the intrathymic vessels. Barely visible in infants, these PVS enlarge with age [156]. At the CMJ, they are the entry sites for both immature lymphoid progenitors and recirculating T-, B- and dendritic cells from the blood and for exit for mature T-cells to the blood [145, 157, 158]. Furthermore, they are niches for B-cells and plasma cells spontaneously secreting protective antibodies that also prevent tolerance to viruses in healthy subjects [159], as in mice [160], or secrete pathogenic antibodies in early-onset myasthenia gravis (EOMG; see below).

Successfully selected CD69^low^ nascent T-cells emigrate from the medulla to the PVS to the blood. That depends partly on cytokines, chemokines and integrins, also on (a) the sphingosine-1-phosphate (S1P) gradient between the S1P^low^ medulla and the S1P^high^ blood [43], the balance between S1P-production by pericytes in the PVS and degradation by stromal cells (mostly DCs) in the medulla in mice [157, 161–164] and humans [165], and (b) the corresponding upregulation of S1P receptors (S1PR_1_) on the nascent T-cells; (c) signals from endothelial cells to pre-emigrant T-cells [166], and T-cell intrinsic factors like the protein kinase MTS1 (a member of the Hippo pathway) and the actin regulator, Coronin-1A (encoded by *CORO1A*) that regulate T-cell polarisation, adhesion and migration [157].

B-cells in PVS accumulate progressively with age and switch from a mainly IgM^+^ IgD^+^ CD27^--^ naïve phenotype in infants to a class-switched IgG1/IgG3/IgA^+^ CD27^+^ memory phenotype in adults [159].

### Human autoimmune diseases related to T-cell migration and the PVS

Defects of thymocyte adhesion, migration and egress from the thymus are typically associated with a combined (T-/B-cell) immunodeficiency, as exemplified by mutations of *MST1* [167–169] and *CORO1A* [163]. Poor adhesion and migration that compromise interactions between thymocytes and antigen-presenting cells attenuate positive and negative selection and the development and function of Tregs [43, 169, 170]. In *MTS1* mutant thymi, some T-cells typically escape to the periphery, where rarely oligoclonal or even monoclonal lymphoproliferations, organ infiltrates and autoantibody-mediated cytopenias develop [168, 171]. In *CORO1A* mutations, the egress defect is generally so severe that autoimmunity is generally prevented.

### Pathology

Thymi with defects in egress due to mutations of *MTS1* and *CORA1A* usually retain their corticomedullary architecture [163]. The generally mild defect in *MTS1* mutations shifts the balance towards a higher proportion of mature thymocytes, while the massive block to egress in *CORO1A* mutated thymi leads to ‘giant PVS’ with accumulations of mature T-cells [172].

## Autoimmune myasthenia gravis—the inflamed and neoplastic thymus

Myasthenia gravis (MG) is a CD4^+^ T-cell-dependent HAID, where autoantibodies interfere with neuromuscular transmission, causing muscle weakness. Autoantibodies in 80% bind to the AChR [173]. This ‘AChR-MG group’ comprises EOMG (onset before age ~50), late-onset MG (LOMG; onset age >50) and thymoma-associated MG (TAMG) that show inflammatory, atrophic and neoplastic thymic alterations, respectively, with distinct clinical and genetic associations (Table 1). Here, we focus an EOMG and TAMG, since patients with LOMG appear heterogeneous, pathogenesis is largely unclear [175, 180, 181], and MG types due to other autoantibodies have an uncertain thymic phenotype [182–184].

**Table 1:** Features and risk factors of myasthenia gravis (MG) subtypes with anti-acetylcholine receptor (AChR) autoantibodies, comprising early-onset MG (EOMG), late-onset MG (LOMG) and thymoma-associated MG (TAMG) [82, 174–180]. Onset-ages may be subject to revision. EOMG and LOMG may prove to overlap, and the cut-off age(s) to differ between the sexes

MG type	Autoantigen targets	Onset-age (years)	M:F ratio	Genetic risk factors in Caucasians	Thymic pathology	AIRE^+^ mTECs	Myoid cells
EOMG	AChR	<50–60	1:3	MHC class I >II^a^ (*CHRNA1*^b^) *PTPN22* (*CTLA4*^*low*^) (*TNIP1*)	Ectopic germinal centres	Normal number	Normal number
LOMG	AChR ± titin cytokines RYR1/2	>50–60	2:1	MHC class II >I^a^ *TNFRS11A* (*PTPN22) (CTLA4*^*low*^	Atrophy	Reduced	Reduced
TAMG	AChR ± titin cytokines RYR1/2	Any (median age ~50)	1:1	None established (*CTLA4*^*high*^)	Thymic epithelial neoplasm	Absent	Absent

*CHRNA1* AChR *a*-subunit gene [82], *PTPN22* protein tyrosine phosphatase, non-receptor-type, 22, *CTLA4* cytotoxic T lymphocyte-associated 4: the unique CTLA4 high-expresser risk genotype in TAMG suggests a role of CTLA4 in central tolerance failure [176], *TNIP1* TNFAIP3-interactin protein 1, *TNFRS11A* TNF receptor superfamily, member 11A (RANK), *RYR1/2* ryanodine receptors 1 and 2

^a^Associations awaiting confirmation [174–177, 179, 180] are given in brackets; the HLA-DQA1*05:1 gene is predisposing in EOMG and protective in LOMG [177, 180]

^b^Cytokines (type I interferons; IL12)

### Thymic inflammation and immunopathogenesis of early-onset MG

**Fig. 2:**
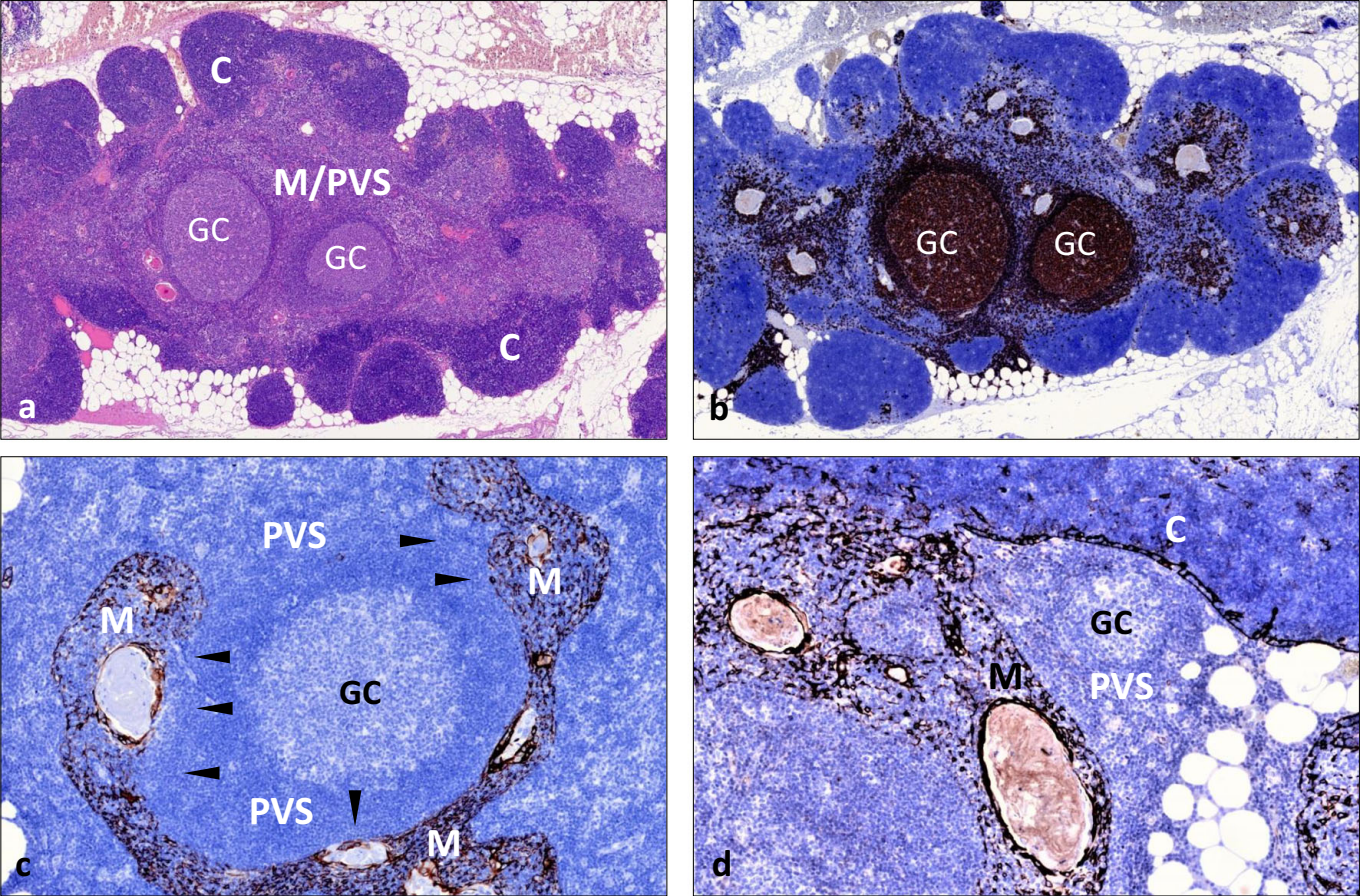
Thymus with ‘thymic lymphoid follicular hyperplasia’ (TFH) in early-onset myasthenia gravis (EOMG). **a** Hematoxylin-eosin stain showing well delineated dark staining cortical areas (C) and extended, light-staining areas with a merger of medulla and perivascular space (PVS) including two lymphoid follicles with germinal centres (GC). **b** CD20 stain highlights massive increase of B-cells. **c** Keratin 19 stain highlights keratin 19(+) medullary areas (M) compared with a massively enlarged, epithelial-free perivascular space (PVS) with a large lymphoid follicle with germinal centre (GC); interrupted epithelial layer between PVS and medulla (arrowheads). **d** Small lymphoid follicle restricted to the slightly expanded PVS with *intact* continuous epithelial layer between PVS, medulla (M) and cortex (C). Immunoperoxidase

**Fig. 3:**
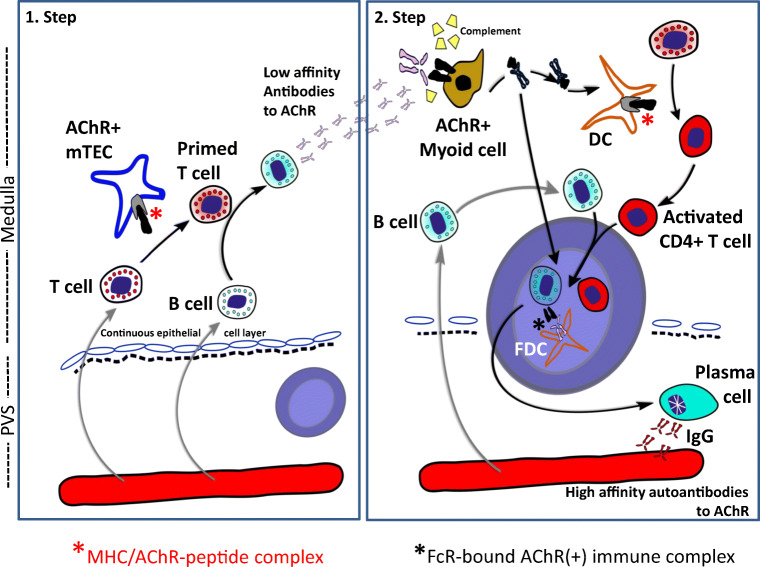
Two-step intrathymic pathogenetic model of early-onset myasthenia gravis. Step 1: On re-entry of acetylcholine receptor (AChR)-reactive T-cells from the blood to the thymus, the T-cells (activated by unknown triggers) get ‘primed’ by medullary thymic epithelial cells (mTECs) expressing MHC/AChR-peptide complexes. The primed T-cells activate thymic B-cells to produce low-affinity anti-AChR antibodies. Step 2: These autoantibodies bind to thymic myoid cells (TMCs) expressing native AChRs, activate complement and induce the release of AChR/antibody complexes from TMC for processing by nearby dendritic cells (DCs) that bind to follicular dendritic cells (FDCs). The germinal centre (GC) reaction finally results in plasma cells producing high-affinity anti-AChR autoantibodies. It is unknown whether lymphoid follicles arise primarily in the PVS (as shown on the left and in Fig. 1d) or in the medulla, and why AChR-reactive T-cells occur very commonly in the ‘physiological’ T-cell repertoire of healthy humans

The hallmark of EOMG is thymic follicular hyperplasia (TFH), i.e. ectopic lymphoid follicles in PVSs merging with the thymic medulla [185] (Fig. 2). TFH shows germinal centres and increased numbers of B-cells and plasma cells and correlates with intrathymic production of heterogeneous IgG autoantibodies with high affinities for native AChR. Female gender and the HLA-DR3 B8 A1 haplotype are strong risk factors [186], B8 appearing the strongest [177], though roles of other loci are less clear (Table 1). EOMG is highly heritable [179] and commonly associated with other AIDs, predominantly thyroiditis, SLE and RA [184, 186]. The triggers of TFH are unknown [187]. A type I interferon signature in the inflamed thymus hinted at viral infections [188], but no specific pathogen could be linked to MG [187]. Still, there are strong arguments for intrathymic initiation of TFH [189, 190]: (1) anti-AChR autoantibodies are preferentially produced in the EOMG thymus [191] by terminal plasma cells [192]; (2) in many patients, they preferentially recognise foetal AChRs [193] that are almost exclusively expressed on thymic myoid cells (TMCs); (3) TMCs are attacked by autoantibodies and complement in EOMG [182, 190] and closely associate with DCs that supposedly cross-present TMC-derived AChR-peptides to autoreactive T-cells for subsequent stimulation of autoreactive B-cells [194]; (4) lymphoid follicles disrupt the normally continuous basal membrane and epithelial cell layer around PVS, displacing TMCs from the tolerogenic medullary parenchyma into the inflamed vicinity of lymphoid follicles, many of which harbour AChR-autoreactive B-lineage cells in germinal centres [192, 195]; (5) in EOMG, mTECs that express unfolded AChR subunits [101] are attacked by complement and anti-epithelial autoantibodies of unknown specificity [196, 197] and over-express CXCL13 that recruits peripheral B-cells to the thymus [198]. Together with the beneficial effect of thymectomy [199], these findings support the ‘intrathymic pathogenesis concept’ of EOMG [189, 200]. Based on these observations, the finding of AChR-autoreactive, CD4^+^ effector T-cells in the repertoire of almost everybody [201, 202] and the fact that mature human T-cells recirculate to the thymus [203] currently favour a 2-step intrathymic pathogenesis model of EOMG [190] (Fig. 3):

Step 1: AChR-reactive T-cells are primed (for unknown reasons) by mTECs that express MHC/AChR-peptide complexes, and then elicit low-affinity antibodies against linear AChR epitopes.

Step 2: These ‘early antibodies’ attack AChR^+^ TMCs, activate complement and cause the release of AChR/immune complexes that, in turn, activate DCs to initiate ectopic follicle and germinal centre formation and focus the autoantibody response onto AChR rather than other muscle targets [190]. It is unknown whether follicles develop primarily near TMCs in the medulla and subsequently in B-cell niches in PVS [159] or vice versa. In either case, a very similar scenario has since been proposed for the development of thymic B-cell follicles in type 1 diabetes-prone NOD mice, including the attack of anti-epithelial autoantibodies on autoantigen (insulin)-expressing mTECs, activation of autoreactive T-cells and accumulation of B-cells [204].

TFH responses might be self-perpetuating in EOMG if (i) AChR persists on TMCs that are damaged by complement but do not disappear [205]; (ii) Tregs are functionally compromised [206, 207]; and (iii) TLR-expressing antigen-presenting cells are abnormally active [208]. Finally, autoreactive T- and B-cells spread to the periphery [209], where, hypothetically, the flow of skeletal muscle-derived AChR/antibody-complexes to regional lymph nodes and functionally impaired Tregs perpetuate EOMG even after thymectomy [184, 199].

### Thymoma and the immunopathogenesis of TAMG

Thymomas are thymic epithelial tumours with variably mixed cortical and medullary differentiation accompanied by thymopoiesis in >90% of patients [210, 211]. TAMG is the single most common thymoma-associated HAID (30–40%), while others (e.g. thyroiditis, RA, and especially SLE, pure red cell aplasia (PRCA), hypogammaglobulinaemia or other bone marrow failures are individually less common (each 1–5%) but, together with TAMG, amount to over 50% thymoma-associated HAIDs [18]. Most of the HAIDs are CD4^+^ T-cell-dependent, autoantibody-mediated (e.g. TAMG), while cytotoxicity may be operative in others (e.g. in PRCA) [212, 213]. Unlike in EOMG, >80% of patients with thymomas have autoantibodies to non-AChR skeletal muscle antigens (titin and RYRs) and others that neutralise such cytokines as type I interferons (~70% [22, 61, 62] and IL-12) (Table 1). Those against the muscle have been attributed to the lack of thymic myoid cells (TMCs) in thymomas [18] and/or expression of AChR, titin and RYR epitopes in neoplastic thymic epithelial cells [100, 214].

The autoantibodies against type I interferons (all 12 subtypes [190]) are among several striking parallels with >90% of APECED patients: others include the chronic mucocutaneous candidiasis (CMC) that is often the first sign of APECED, also occurs in ~3% of thymoma patients and has an autoimmune basis in both—i.e. autoantibodies against IL-17s and/ or IL-22 and loss of the cytokine-producing cells [62]. The apparent absence of AIRE in most thymomas [215] renders these tumours the most practical alternative for studying AIRE-deficient thymopoiesis in humans [22, 216]. The differences between these syndromes include the rarity in APECED patients of MG or of almost any neurological disorder or autoantibody [22]; they may partly reflect the contrasting effects of *AIRE* mutations present since conception in APECED versus the focal acquisition of a neoplastic AIRE-deficient clone of thymic epithelial cells in adult thymoma patients who already have an established normal peripheral immune repertoire. The clinical variability among thymoma patients has given clues to the pathogenesis of TAMG [18]:

1. Strong gender and genetic risk factors apparently contribute little to its development (Table 1), suggesting that the tumour is its main etiological factor.
2. Strongly thymopoietic thymomas confer greater TAMG-susceptibility than those with poor thymopoiesis; thymic carcinomas without thymopoiesis almost never develop MG [214].
3. Thymomas that generate naïve CD4^+^ T-cells that then contribute to the peripheral TH-cell repertoire associate more strongly with TAMG than others that fail to ‘export’ single positive CD4^+^ progeny [212, 217]. Also, TAMG(+) thymomas appear enriched for AChR-reactive thymocytes [218]. The reason for this dichotomy at the level of CD4^+^ T-cells is only partly understood [219]. By contrast, export of CD8^+^ T cells from thymomas is maintained irrespective of MG status [217].
4. In sharp contrast with the findings in EOMG thymi [191], conformation-specific autoantibodies to AChRs are not produced by cells from thymomas [100, 220, 221]. However, other autoantibodies against IFN-αs or IL-12 are produced by thymoma plasma cells; moreover, their titres usually rise sharply when thymomas recur, suggesting immunisation against linear epitopes within the tumours [221].
5. With rare exceptions [22, 222], levels of mRNA encoding the AChR α-subunit are higher in TAMG(+) thymomas than in TAMG(−) thymomas, hinting at immunisation there rather than tolerance induction [22, 100, 214, 221], unlike in the normal thymus [82]. Similarly, IFN-αs are present in thymomas [190], obviously AIRE-independent and clearly fail to tolerise there—again contravening standard dogma [14]. AIRE reportedly has additional tolerogenic actions [223]. If so, their loss may create aberrant thymic environments where it becomes ‘dangerous’ to express autoantigens. Wolff et al. [22] therefore proposed two parallel mechanisms in APECD thymi: in one, T-cells are actively auto-immunised, exported and go onto attack early, especially causing the unusual TH17 cell, parathyroid and adrenocortical failures, which mostly appear by ages 5–10 (in ~90 to ~70% of patients); in another scenario, T-cells simply fail to get tolerised, e.g. versus insulin, which happens randomly, much later and less frequently.

**Fig. 4:**
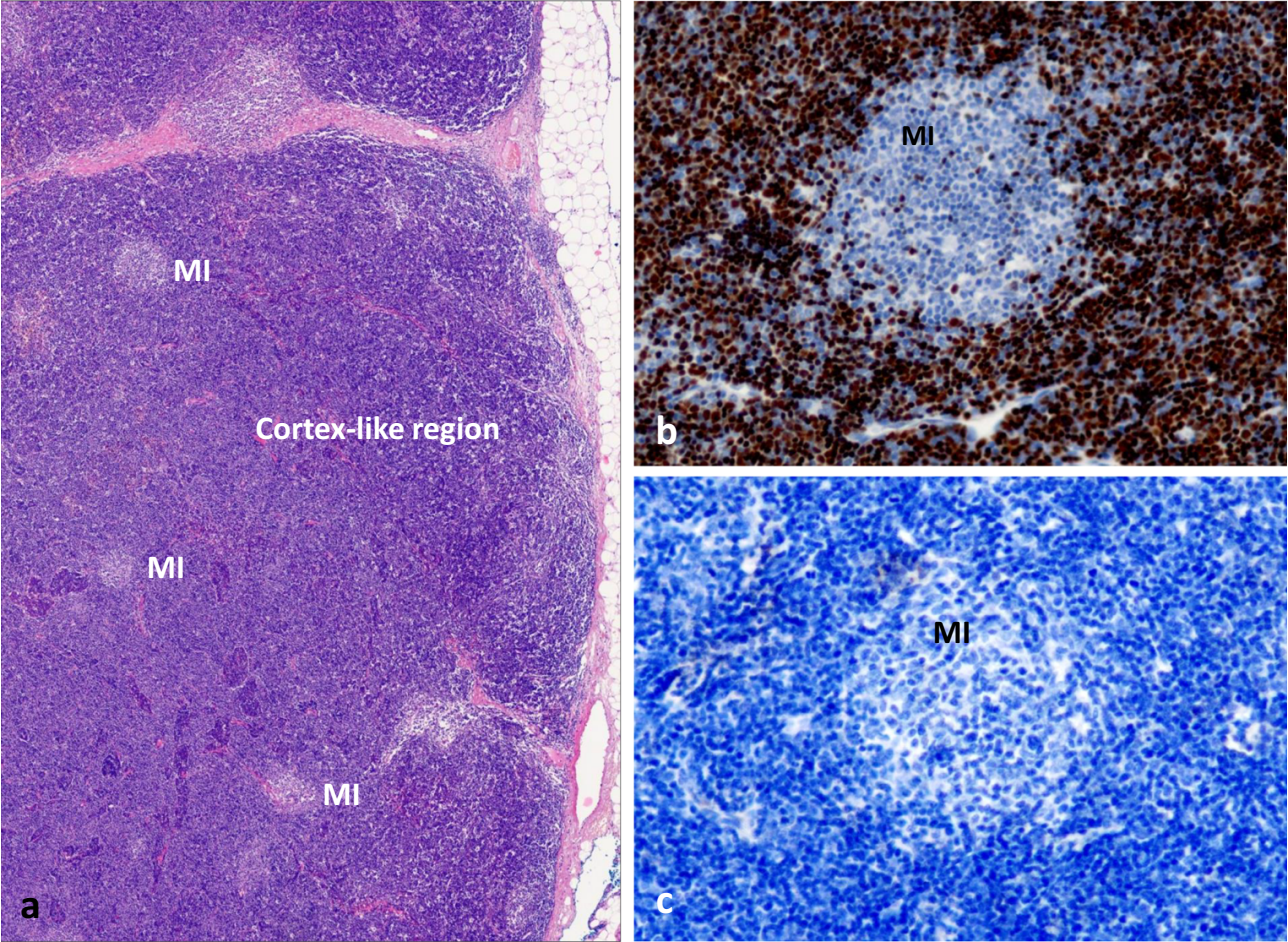
Typical abnormalities of a thymoma with extensive thymopoiesis. **a** Conventional hematoxylin-eosin stain with predominant (dark) cortical areas and tiny (light staining) medullary regions. **b** TdT stain highlights extensive positively stained cortical areas (C) and small, unstained ‘medullary island’ (MI). **c** Absence of B-cells throughout the tumour (PAX5 stain). Note absence of Hassall corpuscles (due to absence of AIRE expression, not shown ). Immunoperoxidase

The following abnormalities in thymomas could, in theory, contribute to the development of TAMG, but surprisingly are also common in TAMG(−) thymomas [18, 210, 211, 215, 224–226]: the frequently reduced expression of MHCII antigens on TECs; the common MHC haploinsufficiency of TECs due to loss of 6p21; the reduced expression of proteases in cTECs (e.g. PRSS16); the reduced size of medullary compared with cortical areas; the lack of AIRE^+^ mTECs and of Hassall corpuscles; the defective generation of FOXP3^+^ Tregs in thymomas; the paucity of B-cells and TMCs (Fig. 4).

Together, the findings have suggested a 4-step pathogenetic model for most thymomas that show thymopoiesis and express AChR/Titin epitopes:

1. Biased positive selection of developing specific TH-cells by neoplastic linear AChR/titin peptide-overexpressing TECs with cortical features expressing reduced levels of some HLA variants [211, 214, 224]
2. These self-reactive TH-cells survive or are even pre-primed *in situ* by their target autoantigens, partly because of the absence of AIRE^+^ mTECs and Tregs [190], also because of combined defects of medullary functions (including lack of myoid cell-derived AChRs and titin for tolerogenic cross-presentation by APCs)
3. Autoreactive mature TH-cells pass the bottleneck to terminal maturation [217], and escape apoptosis in thymomas [219], exit to the blood and ‘infiltrate’ the existing tolerant T-cell repertoire with thymoma-derived autoreactive T-cells [212, 218]
4. In the periphery [209], including the remnant thymus [221], these escaping autoreactive TH-cells stimulate B-cells to generate autoantibodies against native AChR after appropriate stimulation [217]. Once initiated, skeletal muscle-derived AChR/autoantibody complexes presented in regional lymph nodes perpetuate TAMG even after thymoma removal [227].

For the rare thymomas without thymopoiesis and AChR/Titin expression, alternative pathogenetic models may apply [18].

Finally, one should not forget that thymomas are malignant tumours that often require oncological interventions. Due to their propensity to ‘poison’ the immune system with potentially autoreactive CD8^+^ and CD4^+^ T cells [217], thymomas are exceptionally risky targets for immune checkpoint inhibitors, since they can unleash severe if not fatal autoreactivities particularly focused on skeletal and cardiac muscle [228].

## Conclusion

Thymic tolerance-inducing mechanisms and their failure are extremely complex and have been difficult to study, particularly in humans, in whom autoimmune syndromes have crucially spotlighted relevant genes and their actions. Novel single cell and spatial transcriptomic approaches, in conjunction with multiplex imaging techniques, have the potential to open new perspectives when applied to normal and diseased human thymus and appropriate mouse models.
